# Detection of *Leishmania donovani* in Wild-Caught Phlebotomine Sand Flies in Endemic Focus of Leishmaniasis in Himachal Pradesh, India

**DOI:** 10.1093/jme/tjab202

**Published:** 2021-12-01

**Authors:** Suman Lata, Gaurav Kumar, V P Ojha, Ramesh C Dhiman

**Affiliations:** ICMR-National Institute of Malaria Research, New Delhi, India

**Keywords:** Leishmaniasis, cutaneous leishmaniasis, sand fly, Himachal Pradesh, *Phlebotomus longiductus*

## Abstract

An endemic focus of cutaneous leishmaniasis (CL) is located in the Sutlej River Valley in Himachal Pradesh (India) in the north-western Himalaya where CL co-exists with visceral leishmaniasis (VL). In areas of the Indian subcontinent such as Rajasthan, cutaneous leishmaniasis is transmitted by *Phlebotomus papatasi* (Scopoli) and *Phlebotomus salehi.* In Himachal Pradesh, *Phlebotomus longiductus* (Parrot) is suspected to be the vector for CL. In the current study, sand flies were collected and tested for *Leishmania* infection and to confirm the vector species causing CL. Sand flies were collected during April and September of 2017–2019 from CL endemic villages of Rampur (Shimla), Nirmand (Kullu) and Nichar (Kinnaur) districts of Himachal Pradesh. The sand flies were identified as *Phlebotomus (adlerius) longiductus* (Parrot) and *Phlebotomus (larrousisus) major (*Annandale). The density of *P. longiductus* was found highest. The elevation of villages ranged from 947 m to 2,130 m and were far from the presence of subsoil water. Field collected sand flies tested positive (7.69%) for *Leishmania donovani* by PCR-RFLP. The *L. donovani* sequences detected from *P. longiductus* were 97% similar to *L. donovani* sequences reported from the cases of CL in Himachal Pradesh.The *Leishmania* positive sand flies were morphologically identified as *Phlebotomus adlerious longiductus* providing one step further evidence towards the vector status of CL in Himachal Pradesh. The findings of the study are of epidemiological significance for strategic planning of vector control for leishmaniasis in India.

The leishmaniases are a group of diseases caused by the protozoan parasite *Leishmania*. Over 20 species of *Leishmania* known to be infective to humans, are transmitted by the bite of infected female phlebotomine sand flies. Of 200 countries and territories reporting to WHO, 97 countries are endemic exclusively for visceral leishmaniasis (VL), 65 for both VL and cutaneous leishmaniasis (CL) while 22 countries are endemic for CL alone (WHO 2017). Historically, the focus of CL in India was reported from Bikaner district in the Thar desert of Rajasthan in 1973. Desert gerbil, *Meriones hurriane* is the reservoir for zoonotic CL and *Phlebotomus salehi* Meshghali is the vector species. Dog has been reported as a reservoir for anthroponotic CL and *Phlebotomus papatasi* as the vector (WHO 2010). After a gap of around 30 years, a new focus of CL was reported along the River Sutlej in Himachal Pradesh from 2003 to 2005, (Sharma et al 2003, Sharma et al 2005). While investigating the focus of CL in Himachal Pradesh, Sharma et al. (2003, 2005) identified *Leishmania tropica* and *Leishmania donovani* as the causative parasite and *Phlebotomus longiductus* was suspected to be the vector. Only one sand fly was found positive for *L. tropica,* and the authors recommended further studies to confirm the vector species responsible for transmission of CL in Himachal Pradesh (Sharma et al. 2009).

Recently Sandhya et al. (2017, 2018) have further studied the clinic-epidemiological features of CL along the Satluj river in districts of Shimla, Kullu, and Kinnaur warranting studies on incrimination of vector species of CL (Sandhya et al. 2017, 2018). Thakur et al. 2018 in a review reported that CL has expanded to several parts of the country like Jammu & Kashmir, Assam, Haryana, Delhi, Uttar Pradesh, and Kerala raising a public health concern. Unless we unravel the epidemiology of CL and VL in Himachal Pradesh or Kerala etc, the goal of elimination of leishmaniasis cannot be achieved. The present study was, therefore, undertaken in reported foci of Shimla, Kinnaur, and Kullu districts of Himachal Pradesh to determine the vector of CL.

## Methodology

### Study Area

The present study was undertaken in Rampur, Nirmand, and Nichar subdivisions of Shimla, Kullu, and Kinnaur districts of Himachal Pradesh located along the Sutlej River Valley between 31°05′0″N and 31°42′0″N latitude and 76°52′E and 78°24′0″E latitude. Altogether 16 villages were selected for collection of sand flies (Lata et al. 2021) based on accessibility.

### Collection of Sand Flies

The collection of sand flies was made during April and September 2017–2019. Sand flies were collected between 06:00–09:00 h.from human dwellings and cattle shades using aspirator, sticky trap, and CDC light trap. The density of sand flies collected from indoor resting sites was expressed as Man Hour Density (MHD) (Srivastava and Dhariwal 2016). After collection, the sand flies were stored in 70% ethanol for identification. Head and last three abdominal segments with external genitalia were used for morphological identification and remaining abdominal segments were used for DNA extraction.The specimens were kept in 10% Potassium hydroxide solution at room temperature for 2h before mounting on slides. Identification of sand flies was done following the identification key by Lewis (1978) and Kalra and Bang (Kalra et al. 1988).

### DNA Extraction, Polymerase Chain Reaction—Restrictiction Fragment Length Polymorphism (PCR-RFLP) and DNA Sequencing

Total DNA was extracted from 52 bloodfed sand flies individually using the Qiagen DNA mini kit (Qiagen), as per manufacturer’s instructions, and eluted in 20 µl buffer. The details of DNA extraction, PCR-RFLP, and DNA sequencing are given in Lata et al. (2021)

## Results

### Man Hour Density of Sand Flies

The sand flies collected from various villages were morphologically identified as *Phlebotomus (adlerious) longiductus* and *Phlebotomus (larrousisus) major.* The altitude of villages ranged from 947 to 2,130 m in Shimla, Kullu, and Kinnaur districts respectively (Table 1). The highest MHD was found in Kinnaur district (80), followed by Kullu (54), and Shimla district (15).

**Table 1.** Village wise collection of Phlebotomine sand flies from Rampur (Shimla), Nirmand (Kullu), and Nichar (Kinnaur) district in September and April months during 2017–2019

S.no	Village	Elevation(m)	Sand flies collected	MHD
	Shimla district			
1	Shingla	1249	15	15
2	Sarlaprog	1265	12	12
3	Kalna nogli	1185	8	8
4	Jhakhdi	1175	0	0
5	Rampur	1033	0	0
6	Devton	1011	0	0
	Kullu district			
7	Jagaat Khana	1002	0	0
8	Brow	1015	54	54
9	Randal	1240	35	35
10	Chaati	1077	3	3
11	Thachwa	947	0	0
12	Shoga	1158	0	0
	Kinnaur district			
13	Chagaon	2130	41	41
14	Punang	1775	115	80
15	Kilba	1859	3	3
16	Kasthla Wangtoo	1725	3	3
17	Tangling	1952	2	2
18	Tapri	1673	0	0
19	Urni	2234	0	0
20	Bhawanagar	1545	0	0

### Detection of *Leishmania* in Sand Flies

**Fig. 1.**
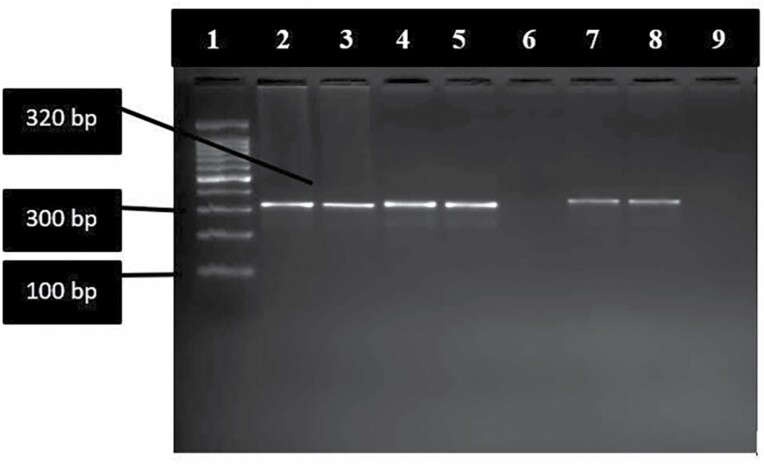
DNA amplification of different *Leishmania* strain and DNA isolates from sandflies. LITSR/L5.8S set amplifies a 320 bp fragment of ITS1 region of *Leishmania* genus-specific DNA. Lane 1:100bp Ladder, lane 2 and 3 sand-fly DNA isolate sample, lane 4: *Leishmania donovani* positive control, lane 5: *Leishmania tropica* positive control, lane 6: negative control, lane 7 and 8 sand-fly DNA isolate.

**Fig. 2.**
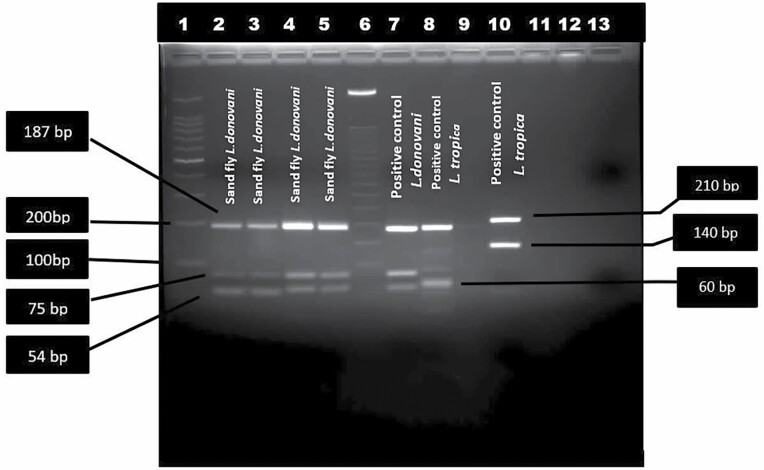
*Leishmania* species-specific restriction fragment length polymorphism –Polymerase Chain Reaction assay of different parasite strain and isolates from sandflies. lane 1: 100 bp DNA ladder; lane 2–5 sand-fly sample; lane 6: 50bp DNA ladder; lane 7: *Leishmania donovani* positive control; lane 8; *L.tropic* positive control; lane 9: Negative control; lane 11*: L. major* positive control.

Total DNA extracted from 52 bloodfed sand flies were subjected to PCR for detection of *Leishmania*, of which four sand flies tested positive for *Leishmania* with both JW11/12 and LITSR/L5.8S *Leishmania* genus-specific set of primers (Fig. 1). By PCR –RFLP, it was confirmed that all four *Leishmania* positive sand flies showed *L. donovani* fragments with bands of size 54 bp, 75 bp and 187 bp (Ranasinghe et al. 2015) (Fig. 2).

### Sequence Analysis

#### *In Silico* Analysis

MN861106.1 and MN861107.1 accession having sequences of *L. donovani* identified from *P. longiductus* showed close similarity with the *L. donovani* sequences from cases of CL in Himachal Pradesh with accession numbers MT423519-21 (Lata et al. 2021). Only a single “T” nucleotide deletion was observed at the 265 nt position in both the sequences (MN861106.1 and MN861107.1) in comparison to the *L. donovani* sequences from the cases of CL in HP (MT423519-21.)

**Fig. 3.**
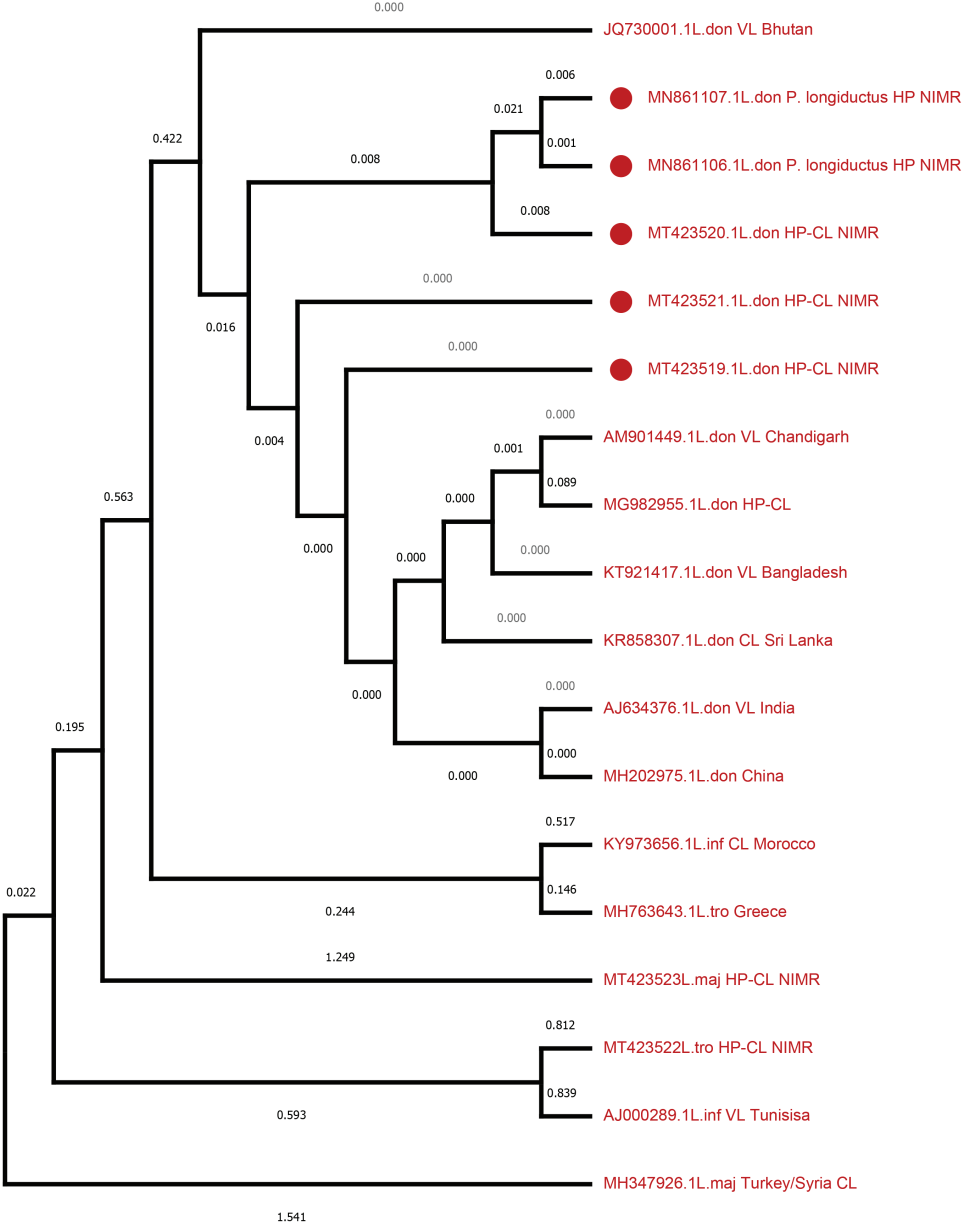
ITS1-based molecular analysis of isolates from *Phlebotomus longiductus* in Himachal Pradesh, India. Phylogenetic tree of ITS1 sequences from test isolates (designated as L. dono P. Longiductus HP NIMR) and standard WHO *Leishmania* strains has been constructed using Neighbor-joining Method in the phylogeny program of MEGAX builder (10.1.8). GenBank accession numbers are indicated.

In the tree obtained by the Neighborhood-joining method (Fig. 3), it has also been revealed that both the accessions MN861106.1 and MN861107.1 were found 97% similar to *L. donovani*. Both of these sequences showed ~45–97% of similarity with the other species included in the analysis. The distantly related species *Leishmania tropica* (MT423522 MH763643), *Leishmania major* (MT423523 and MH347926), and *Leishmania infantum* (AJ000289 and KY973656) were grouped under a distant clade (Fig. 3), indicating about the sequence variability. These species showed similarity of 45% (*L. major*), 75% (*L. tropica*), and 96% (*L. infantum*) with *L. donovani* species. The results of detection of natural infection of *L. donovani* by PCR and DNA sequencing techniques are given in Table 2.

**Table 2.** Detection of *Leishmania* species in sand flies

Village and district	Sand fly species	*Leishmania* species by PCR -RFLP method	Leishmania species by DNA sequencing method
Shingla (Shimla)	*P. longiductus*	*L. donovani*	*L. donovani*
Brow (Kullu)	*P. longiductus*	*L. donovani*	*L. donovani*
Brow (Kullu)	*P. longiductus*	*L. donovani*	*L. donovani*
Kalna Nogli (Shimla)	*P. longiductus*	*L. donovani*	*L. donovani*

**Fig. 4.**
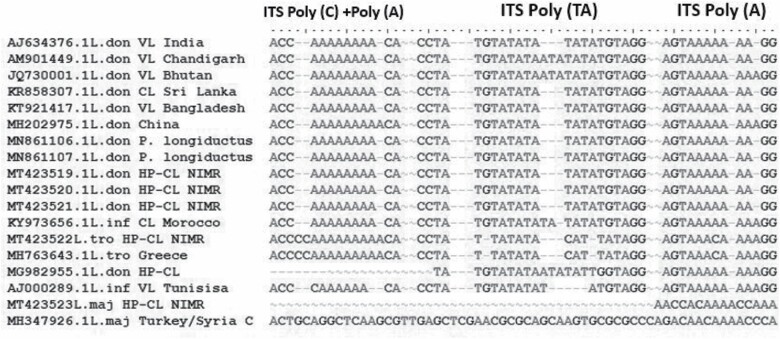
Multiple sequence alignment of ITS1 microsatellite repeat sequences of representative isolates from *Phlebotomus longiductus* and reported from CL cases with those of *Leishmania donovani* complex reference strains from different geographic regions. The evolutionary distances were computed using the Maximum Composite Likelihood method. Sequences were aligned by using BioEdit sequence alignment program.

In order to know both the genetic and geographic relatedness of identified isolates among themselves and with standard reference strains, the results of ITS1 polymorphic microsatellite repeat analysis indicated that *Leishmania* sequences isolated from *P. longiductus* are similar to only *L. donovani* isolates identified from different locations. However, the polymorphism was detected in the microsatellite markers in the sequences derived from either *L. infantum, L. tropica,* or *L. major* species (Fig. 4, Table 3). By sequence analysis it can be concluded that the *L. donovani* sequence detected from CL cases in HP are similar to *L. donovani* sequences detected from *P. longiductus* which provide the one-step further evidence towards the vectorial status of *P. longiductus* for CL in HP and also confirming that the *L. donovani* is the predominant *Leishmania* species detected.

**Table 3.** *Leishmania* species used in ITS-based microsatellite polymorphism and phylogenetic analysis of *Leishmania* isolates identified

GenBank accession	Standard *Leishmania*	Place of origin	Poly C	Poly A	Poly TA	Poly A
AJ000289.1	*L. infantum*	Tunisisa	3	6	4	8
AJ634376.1	*L. donovani*	India	2	8	5	7
AM901449.1	*L. donovani*	Chandigarh	2	8	2,TAA,3	7
JQ730001.1	*L. donovani*	Bhutan	2	8	2,TAA,3	8
KR858307.1	*L. donovani*	Sri Lanka	2	8	5	7
KT921417.1	*L. donovani*	Bangladesh	2	8	5	7
KY973656.1	*L. infantum*	Morocco	2	8	6	8
MG982955.1	*L. donovani*	Himachal Pradesh	Heterogenous		2,TAA,3	8
MH202975.1	*L. donovani*	China	2	9	5	8
MH347926.1	*L. major*	Turkey/Syria CL	Heterogenous			
MH763643.1	*L. tropica*	Greece	4	9	3, CAT,2	3, C, 4
MT423519	*L. donovani*	HP NIMR	2	8	5	7
MT423520	*L. donovani*	HP NIMR	2	8	5	7
MT423521	*L. donovani*	HP NIMR	2	8	5	7
MT423522	*L. tropica*	HP NIMR	4	9	3, CAT,2	3, C, 4
MT423523	*L. major*	HP NIMR	NA			4, CC 4
MN861106.1	*L. donovani*	HP NIMR	2	8	5	7
MN861107.1	*L. donovani*	HP NIMR	2	8	5	7

## Discussion

The findings of the present study reveal that phlebotomine sand flies are found up to the altitude of 2,130 m and their distribution is wide spread in villages in Shimla, Kullu, and Kinnaur districts of Himachal Pradesh. In China, the distribution of *P. longiductus* has been reported from 2,100 m elevation (Guan et al. 2016), however, we found its distribution ranging from 947 to 2,130 m. In study villages close to river Sutlej, the maximum MHD of sand flies was found up to 54 and 16 in villages of Kullu and Shimla district respectively. On the other hand, in village Punang, which is located at an altitude of 1,775 m in Kinnaur district and far away from Sutlej river, the highest MHD was 80. Earlier studies from Himachal Pradesh reported only the number of sand flies collected (did not report MHD) and the distribution of sand flies was also not such widespread as observed in the present study.

Sharma et al. (2009) reported one *P. longiductus* as suspected positive for *L. tropica.* The present study confirms the detection of *L. donovani* from field-collected *P. longiductus* (confirmed by both PCR-RFLP and sequencing methods) from the foci of CL in Himachal Pradesh, India which provide one step further evidence towards vectorial status of *P. longiductus*. Further work on identification of blood meal sources of *P. longiductus* and transmission through artificially infected sand flies to a susceptible host is desired to confirm it as vector of CL.

As per World Health Organization (1990), *P. longiductus* has been listed as a proven vector of VL in Kazakhstan, Kyrgyzstan, and in China, however, in these countries, *P. longiductus* was not found naturally infected with *L. donovani*. The present study areas being close to China border reflect the epidemiological characteristics of CL similar to the China. The endemic areas of VL and CL in India are diverse, i.e. VL is in eastern part of the country while CL is mainly in Rajasthan and Himachal Pradesh. In the present study area, the parasite species, i.e., *L. donovani* have been detected (similar to Mediterranean region).

It is indeed intriguing that *L. donovani* has been detected from the cases of CL from several parts of the world including neighboring country Sri Lanka (Siriwardana et al. 2007; Ranasinghe et al. 2015, Thakur et al. 2020). A recent study by Thakur et al. (2020) provided evidence that *L. donovani* isolated from CL patients in Himachal Pradesh is *L. donovani* zymodeme MON37 strain which does not cause VL.

The distribution of *P. longiductus* has also been reported from Banihal and Punch district of Jammu & Kashmir (Lewis 1978), where cases of CL have been reported, however, the parasite and vector species still remain unknown, warranting further studies (Rather et al. 2021). In the geographic belt ranging from Jammu & Kashmir to Himachal Pradesh, a few cases of VL have been reported along the Ravi River in Chamba district but the vector species is unknown (Raina et al. 2016). It is expected that *P. longiductus* may be the vector of VL also as the same species has been reported from Chamba and Kangra districts of Himachal Pradesh (Lewis 1978).The question that still remains unresolved is whether the so-called CL cases detected in Himachal Pradesh are of Dermal leishamanoid (a form of VL) or the pathogenicity of *L. donovani* and *L. tropica* are interchangeable between visceral to cutaneous and vice versa. The sand flies were identified based on morphology. Molecular tools like sequencing and DNA barcoding may be employed to find out the lineage of the species.

The state of Himachal Pradesh had reported cases of VL during 1979 (Naik et al. 1979), 1985 (Dutta et al. 1984) and 2016 (Raina et al. 2016) also but since 2013 there are no reports (www.nvbdcp.gov.in). In Himachal Pradesh both CL and VL both are prevalent but the strain causing VL (zymodeme MON2) has not been detected so far. As there is no surveillance mechanism for detection of VL or CL cases in Himachal Pradesh, it is quite likely that the VL cases are not reported/captured. The cases of CL are also reported passively. On the other hand, the detection of *L. donovani* in field-collected *P. longiductus* in the present study also provides clue that in addition to CL, *P. longiductus* may be vector of VL in Himachal Pradesh as this species is vector of VL in adjacent country, China. Therefore, extensive surveys are desired in Himalayan region of Uttarakhand, Himachal Pradesh, and Jammu and Kashmir to find out the prevalence of VL and the vector species. The goal of elimination of Kala-azar from India, the validation of which was done in 2019 (WHO 2019), may be difficult to achieve unless the Himalayan foci of CL and VL are fully studied.

### Conclusion

The detection of *L. donovani* in field-collected *P. longiductus* from the villages along Sutlej River in Shimla and Kullu districts of Himachal Pradesh provides one-step further evidence towards the vectorial status of *P. longiductus* for cutaneous leishmaniasis in Himachal Pradesh. The findings are of epidemiological significance and strategic planning of vector control for leishmaniasis in India. The need of the hour is to protect the population from the bites of sand flies, therefore, communities in the affected areas should be educated to prevent themselves from the bites of sand flies.

## Data Availability

The datasets used and/or analyzed during the current study are available from the corresponding author on reasonable request.
